# Spatial Accuracy of Predictive Saccades Determines the Performance of Continuous Visuomotor Action

**DOI:** 10.3389/fspor.2021.775478

**Published:** 2022-01-17

**Authors:** Chisa Aoyama, Ryoma Goya, Naofumi Suematsu, Koji Kadota, Yuji Yamamoto, Satoshi Shimegi

**Affiliations:** ^1^Graduate School of Frontier Biosciences, Osaka University, Osaka, Japan; ^2^Graduate School of Medicine, Osaka University, Osaka, Japan; ^3^Research Center of Health, Physical Fitness, and Sports, Nagoya University, Nagoya, Japan; ^4^Center for Education in Liberal Arts and Sciences, Osaka University, Osaka, Japan

**Keywords:** eye movement, visuomotor, continuous visuomotor action, time constraint, online control

## Abstract

In a table tennis rally, players perform interceptive actions on a moving ball continuously in a short time, such that the acquisition process of visual information is an important determinant of the performance of the action. However, because it is technically hard to measure gaze movement in a real game, little is known about how gaze behavior is conducted during the continuous visuomotor actions and contributes to the performance. To examine these points, we constructed a novel psychophysical experiment model enabling a continuous visuomotor task without spatial movement of any body parts, including the arm and head, and recorded the movement of the gaze and effector simultaneously at high spatiotemporal resolution. In the task, Gabor patches (target) moved one after another at a constant speed from right to left at random vertical positions on an LC display. Participants hit the target with a cursor moving vertically on the left side of the display by controlling their prehensile force on a force sensor. Participants hit the target with the cursor using a rapid-approaching movement (rapid cursor approach, RCA). Their gaze also showed rapid saccadic approaching movement (saccadic eye approach, SEA), reaching the predicted arrival point of the target earlier than the cursor. The RCA reached in or near the Hit zone in the successful (Hit) trial, but ended up away from it in the unsuccessful (Miss) trial, suggesting the spatial accuracy of the RCA determines the task's success. The SEA in the Hit trial ended nearer the target than the Miss trial. The spatial accuracy of the RCA diminished when the target disappeared 100 ms just after the end of the SEA, suggesting that visual information acquired after the saccade acted as feedback information to correct the cursor movement online for the cursor to reach the target. There was a target speed condition that the target disappearance did not compromise RCA's spatial accuracy, implying the possible RCA correction based on the *post-*saccadic gaze location information. These experiments clarified that gaze behavior conducted during fast continuous visuomotor actions enables online correction of the ongoing interceptive movement of an effector, improving visuomotor performance.

## Introduction

In a table tennis rally, players continuously perform a visually driven action that aims a fast-moving ball from various positions on their rackets within a limited time. The performance is determined by several processes, from the acquisition of visual information to the motor output. In particular, gaze control (eye movement) is an important step that contributes to the selection of visual information to be collected and determines the content to be processed.

Previous studies examining the gaze behavior of athletes during ball sports, such as cricket, tennis, and table tennis, reported that players do not keep their gaze on the moving ball through the whole trajectory. In general, smooth pursuit eye movement following the ball occurs after the ball is released from the opponent, and then predictive saccades often approach the predictive ball's reach positions in advance of the ball (Bahill and McDonald, 1983; Ripoll et al., 1987; Land and McLeod, 2000; Mann et al., 2019). The study on cricket batters found that professional batters make predictive saccades to the ball's bouncing position earlier than amateur batters, indicating saccades are related to batting performance (Land and McLeod, 2000). However, there are no reports that directly demonstrates that saccade performance contributes to physical performance such as striking ball. Moreover, it remains unknown which information related to the saccades is used for the motor control, since saccades provide at least three types of information including visual information from the retina, proprioceptive information from ocular muscles, and an efference copy of the motor command (Sherrington, 1918; Wilmut et al., 2006; Spering et al., 2011).

In the situation of real sports, the visuomotor performance is determined by not only the visual system but also the motor system. In particular, table tennis players are required to make a fast muscular contraction and an execution of accurate motor control in a short time ranging from 0.36 to 0.42 s (Yuza et al., 1992). The fast muscular contraction needs the recruitment of motor neurons, but this recruitment can compromise the movement accuracy due to increased muscular noise (Schmidt et al., 1979; Harris and Wolpert, 1998; Heitz, 2014). Thus, it is difficult to examine how eye movement contributes to the accuracy of visuomotor control in physical movements with fast muscular contractions. More specifically, the role of gaze behavior in fast visuomotor actions to a moving target has not been studied well.

The direct contribution of eye movements to visuomotor actions, especially continuous actions, such as those in table tennis, have hardly been examined. Those that did study this contribution measured gaze direction when striking a ball in table tennis and cricket (Ripoll et al., 1987; Land and McLeod, 2000; Mann et al., 2019). Since the gaze movement data included both eye and head movements, which operate in a compensatory manner, it has been difficult to separately evaluate whether and how eye movements directly contribute to motor performance. Moreover, evaluating the direct relationship between eye and physical movements is even more difficult during continuous and high-speed visuomotor actions in a head-free condition, such as rally in table tennis.

Based on the findings of previous research, we constructed the following two hypotheses (1) Predictive saccades is involved in continuously striking a fast-moving ball (target) under tight time-constrained conditions such as table tennis, and (2) the performance of the saccade contributes to the success or failure of the striking and the accuracy of the strike performance. The purpose of this study was to test these hypotheses and elucidate the role and mechanism of predictive saccades in improving the accuracy of visuomotor reactions.

Therefore, in this study, we developed a novel psychophysical continuous visuomotor task, in which subjects hit a target with a virtual effector by controlling a prehensile force. This task requires no spatial bodily movement, which decreases the influence of muscular factors such as muscle noise. Moreover, the task was performed under a head-fixed condition to examine the direct relationship between the eye movement and task performance. The task was designed to require the basic visuomotor actions needed during table tennis rallies to examine the role of eye movement in the continuous visuomotor action by the players.

## Materials and Methods

### Experiment 1

#### Participants

Twenty-one (nine females and 12 males, aged 21.5 ± 2.0 years) people participated in Experiment 1. All participants were right-handed and had normal or corrected-to-normal visual acuity. The study was approved by the ethics committee of the Graduate School of Medicine, Osaka University, and was conducted in accordance with the Declaration of Helsinki. Informed consent regarding the aim and experimental protocols of the present study was obtained in writing from all participants before taking part in Experiment 1.

#### Experimental Apparatus and Stimuli

**Figure 1:**
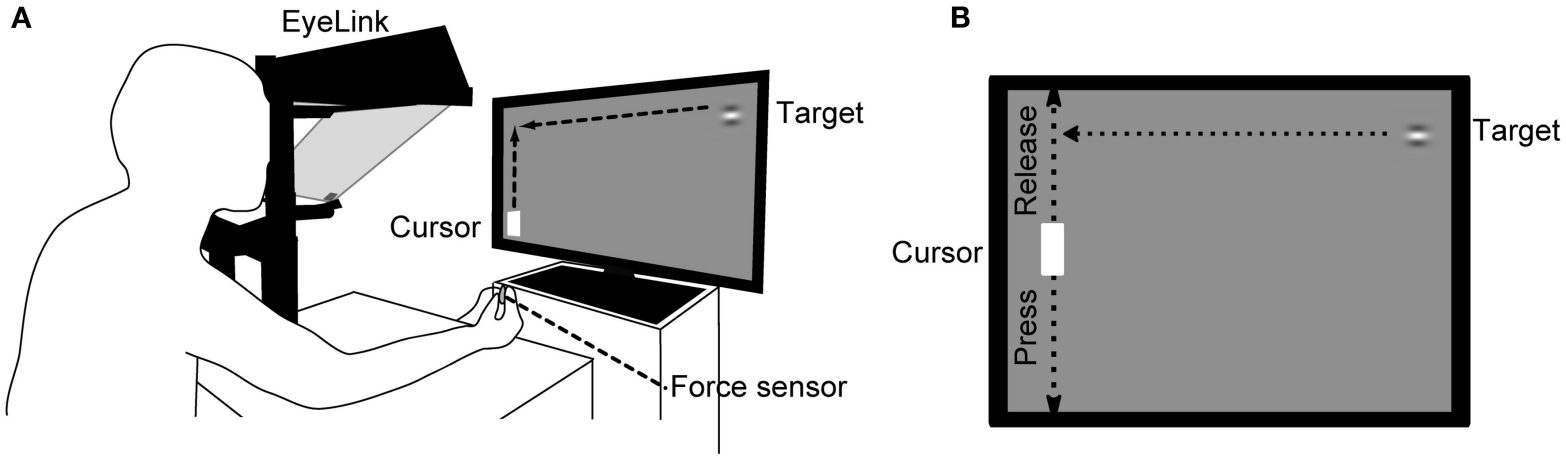
Schema of the experimental setup. **(A)** The participant places his/her head on a chin rest 57 cm in front of the LC display and prehends a force sensor with the thumb and index finger of the right hand. The force-sensor output determines the cursor movement in the vertical direction on the left side of the display. Eye movements are recorded using an eye-tracking system during the task. **(B)** The cursor is positioned at the top of the display when the force sensor is not pressed and moves down in proportion to the force given to the sensor. Target speeds used were 1,000, 2,000, 3,000, 4,000, 5,000, 6,000, and 7,000 pixels/s.

The visual stimuli were generated using custom-made software written in Python and PsychoPy and presented on a 27-inch LC display (Iiyama ProLite G2773HS-GB2; Japan; refresh rate, 144 Hz; mean background luminance, 30 cd/m^2^). The stimuli were Gabor patches (target) with horizontal grating (contrast: 50%, diameter: 2° and spatial frequency 1.5 cycles/° of visual angle) and moved horizontally from right to left in a uniform motion at various vertical positions on the display (Figure 1). The targets appeared one after another, with a new target appearing just after the previous target was hit by the cursor or reached the left edge of the display. The vertical range in which the distance between the target and cursor centers was less than the sum of the target radius and half the vertical length of the cursor was called the “Hit zone”. The target speeds were 1,000, 2,000, 3,000, 4,000, 5,000, 6,000, and 7,000 pixels/s, which corresponded to 29, 57, 85, 113, 141, 169, and 198 deg/s in average visual angular speed. The angular speed was calculated by dividing the angle formed by the two vectors from the midpoint of both eyes to the right end of the LCD display and to the left end of the cursor by the target arrival time. The target-arrival times (TATs) defined as the times from the moment of the target appearance to arrival at the horizontal position of the cursor were 1,541, 772, 515, 387, 310, 259, and 222 ms, respectively. In this study, the target speed was determined to cover the time range of ball arrival from when the opponent hits the ball to when it arrives on the basis of the data of Yuza who measured the time between hits during a rally in an international table tennis match (Yuza et al., 1992). Participants were seated comfortably on a chair 57 cm in front of the display. Their head movements were restrained by a chin rest positioned at the center of the Y-axis and two-thirds of the X-axis from the left side of the display. Participants exerted a prehensile force on a force sensor (sampling rate 1 kHz; USL06-H5-50N-D-FZ and DSA-03A, Tec Gihan Co., Ltd., Japan) with the thumb and index finger of their right hand. A white cursor (height 6° × width 3.2°) moved along the vertical axis at the left side of the display in proportion to the prehensile force. Participants' eye movements during the task were binocularly recorded by an eye-tracker (EyeLink 1000/Tower, SR Research, Canada) at 500 Hz. The left eye movement to the Y-axis direction was analyzed in this study.

#### Continuous Visuomotor Task (CVM Task)

Participants were only instructed to hit the target with the cursor, and did not receive any instructions regarding gaze behavior, so they were able to freely direct their gaze anywhere during the task. The Y-axis position of the cursor corresponded linearly to the magnitude of the prehensile force given to the force sensor. The cursor was at the top of the display when only minimal force applied to the force sensor and moved down as the force increased, reaching the bottom when the force was 30 percent maximum value of each participant. In a calibration session prior to the experiment, the baseline and maximal prehensile forces were measured as the forces when the participant just prehend the force sensor without intending to press and when prehended it maximally, *respectively*. And then, a linear function between the Y-axis position of the cursor and the prehensile force was determined for each participant, where the cursor position was set to be at the top of the display with baseline force and at the bottom with 30 percent force of the maximum. The uppermost target was hit with the cursor by decreasing the force to the baseline. The participants were instructed not to move their right arm during the task. The success and failure of each trial were called a Hit and Miss trial, respectively, and the percentage of Hit trials to the total number of trials (Hit rate) was calculated as the task performance. Prior to the experiment, the participants conducted a practice task for familiarization at target speeds ranging from 1,000 to 3,000 pixels/s. Then they performed a continuous visuomotor task in ascending order of seven target speeds in a block design. Since the participants needed a high concentration to conduct the task, the task duration was unified at 30 s for all target speed conditions, and the inter-task interval for 15 s was set to eliminate the influence of fatigue on the task performance as much as possible. All participants performed trials and breaks alternatively. Specifically, they did 18 trials at 1,000 pixels/s, followed by a 15-s break, 37 trials at 2,000 pixels/s, a 15-s break. Therefore, the number of trials for each target speed differed (1,000 pixels/s, 18 trials; 2,000 pixels/s, 37 trials; 3,000 pixels/s, 54 trials; 4,000 pixels/s, 70 trials; 5,000 pixels/s, 86 trials; 6,000 pixels/s, 103 trials; and 7,000 pixels/s, 119 trials).

### Experiment 2

#### Participants

Thirteen (10 females and three males, aged 19.9 ± 2.7 years) people participated in Experiment 2. All participants were right-handed and had normal or corrected-to-normal visual acuity. The study was approved by the ethics committee of Graduate School of Medicine, Osaka University, and was conducted in accordance with the Declaration of Helsinki. Informed consent regarding the aim and experimental protocols of the present study was obtained in writing from all participants before taking part in Experiment 2, who were different from those in Experiment 1.

#### Experimental Apparatus and Stimuli

The apparatus and stimuli were identical to those used in Experiment 1.

#### CVM Task in Combination With a Temporal Target Disappearance

In Experiment 2, the target disappeared at a specific time in the CVM task to clarify the role of visual feedback.

**Figure 2:**
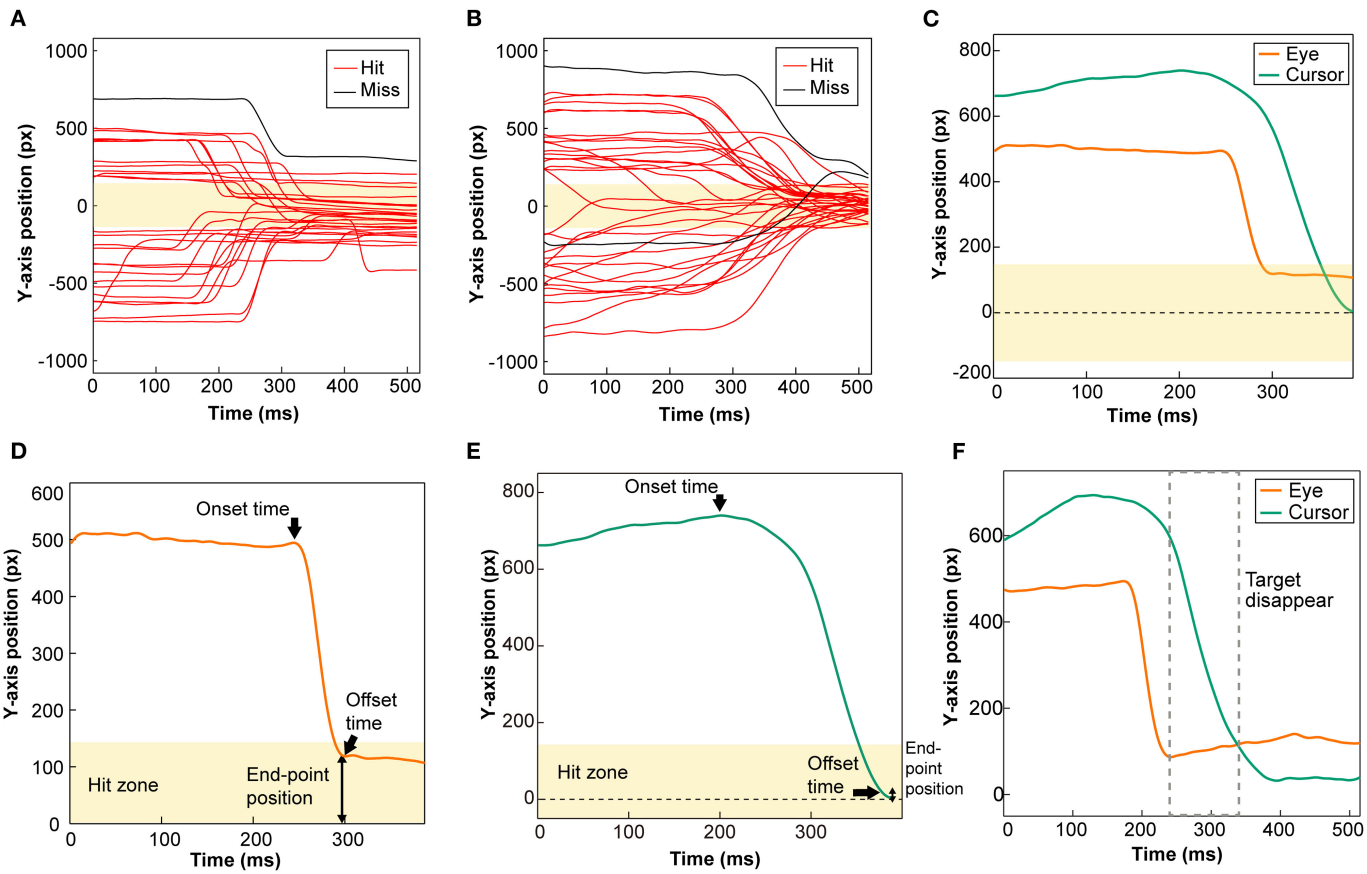
Typical cursor and eye trajectories during the task and analytic parameters of the cursor and eye movement. **(A)** All trial trajectories of eye movements to the vertical axis on the display at the target speed 3,000 pixels/s. **(B)** All trial trajectories of the cursor movement to the vertical axis on the display at the target speed 3,000 pixels/s. Red and black lines indicate Hit and Miss trials, respectively. **(C)** Orange and green lines show a single trajectory of the eye and cursor movement, respectively. **(D)** A single trajectory of eye movement along the vertical axis was used to analyze three parameters (SEA onset time, offset time, and end-point position). **(E)** A single trajectory of cursor movement along the vertical axis was used to analyze three parameters (RCA onset time, offset time, and end-point position). In all figures, the yellow zone indicates the zone in which the cursor hits the target (Hit zone), and the zero value is the Y-axis position of the target. **(F)** Orange and green lines show a single trajectory of the eye and cursor movement at the NoFB condition, respectively. The gray dotted line indicates the time when the target disappeared.

Since Experiment 1 showed that gaze was directed toward the vertical positions of the moving target in a manner dependent on saccadic eye movement (see Figure 2D), we examined the functional role of visual information about the target (no visual feedback condition, NoFB) obtained after the saccade by having the target disappear for 100 ms just after the saccade ended.

During the CVM task, gaze positions were measured separately for the x-axis and y-axis components, and after the velocity vectors on each axis were calculated, the magnitude of the total velocity vector was finally determined in real-time. The target disappeared when the total velocity vector toward the hit zone reached 0. The target reappeared after 100 ms of disappearance. If a trial ends before the target is reappeared, participants were able to know that the next trial has begun by the next target appearance. The results were compared to those without the disappearance (Control).

#### Analyses of Eye Movement and Cursor Movement

The eye movements were analyzed along the vertical axis (Figure 2A). In the present task, the prehensile force given to the force sensor was measured at a 1 kHz sampling rate and transformed to the Y-axis position of the cursor, which was saved for the analysis of the cursor movement (Figure 2B). Data points during eye blinks were replaced with data just before the onset of the blink, and the processed data for eye movement, and the raw data for cursor movement were filtered (fourth-order Butterworth low-pass filter with a 30-Hz cut off and 0-time shift using MATLAB) and aligned with respect to the target position. The trials with no requirement of additional movements, that is, the vertical position of either the gaze or cursor was incidentally within the Hit zone at the beginning of each trial, were excluded from the following data analysis. Figure 2C shows the trajectory of the cursor (green line) and eye (orange line) along the vertical axis of the display in a single trial, in which both rapidly approached the Hit zone (pale yellow area). The approaches of the cursor and eye were called the rapid cursor approach (RCA) and saccadic eye approach (SEA), respectively. SEA occurrence rate was calculated as the incidence percentage of saccades determined from the vertical component of gaze movement relative to the total number of trials.

To quantify how the subject moved the cursor to try to hit the target and what the gaze was doing at that time (Figure 2C), we defined three parameters of the cursor trace line and analyzed the RCA and SEA dynamics in detail: (1) onset time, (2) offset time, and (3) end-point position (Figure 2D). To evaluate the three parameters, we determined the RCA and SEA according to the following analytic procedures. First, we created a velocity profile of the cursor and gaze movements in the Y-axis direction and then extracted all movements toward the Hit zone from the velocity data. On the other hand, since the extracted movement of gaze contains noise, movements that have moved by 5 pixels or more were selected, and the fastest gaze movement was defined as SEA. The start and end times in the RCA and SEA were defined as the tentative onset time and the offset time, respectively. Finally, the onset times for RCA were determined using a threshold of 80 ms, which was the shortest onset latency for visually-triggered body movement reported in previous studies. The visual motion in large- and local fields has been reported to be able to induce eye movements in ultra-short latencies (~80 ms) (Busettini et al., 1997; Masson et al., 2000). In addition, Saijo et al. (2005) reported in the text that there was a participant who had a shoulder flexor muscle EMG response with an ultra-short latency of about 85 ms to a visual stimulus during arm reaching movement. Thus, physical reactions with a latency of 80 ms or more may be stimulus-response and were included in the analysis in the present study. On the other hand, since RCA onset times <80 ms are considered to be incidental rather than responses to visual information, those trials were removed from the following analyses. The percentage of trials in which the RCA occurred with onset time of <80 ms for the total number of trials was 11% at 1,000 pixels/s, 14% at 2,000 pixels/s, 23% at 3,000 pixels/s,33 % at 4,000 pixels/s, 49% at 5,000 pixels/s, 64% at 6,000 pixels/s and 67% at 7,000 pixels/s.

At the time of RCA offset, the Y-axis distance between the centers of the Hit zone and of the cursor was defined as the RCA end-point position and evaluated as the spatial accuracy of RCA. For the spatial accuracy evaluation of SEA, the same one-dimensional evaluation method as RCA and the two-dimensional evaluation method unique to SEA were adopted. Similar to RCA, the distance between the Y-axis between the center of the Hit zone and the gaze at the end of SEA was defined as the SEA end-point position and evaluated as one of the spatial accuracies. As another evaluation of the spatial accuracy of SEA, the distance from the target center position to the gaze position (gaze-target distance) at the end of SEA was calculated.

### Statistical Analysis

**Figure 3:**
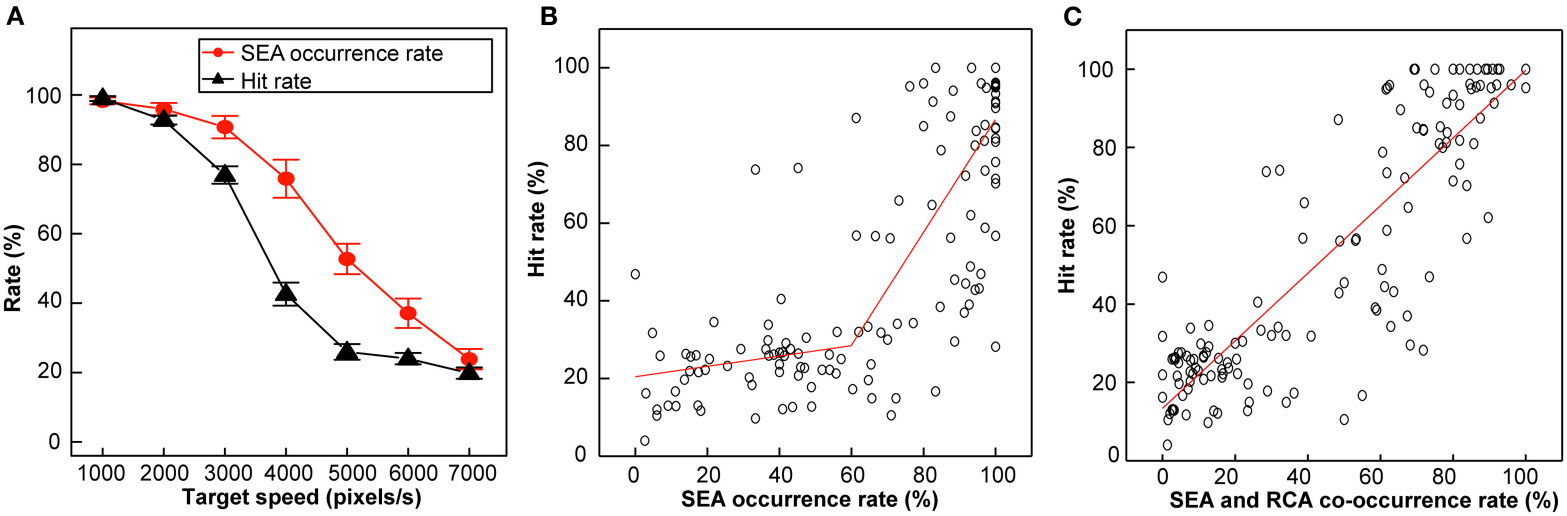
Hit rate, SEA occurrence rate and RCA occurrence rate in Experiment 1. **(A)** SEA occurrence rate (red) and Hit rate (black) plotted against different target speeds. Error bars are SEM. **(B)** Correlation between the Hit rate and SEA occurrence rate. **(C)** Correlation between the Hit rate and SEA and RCA co-occurrence rate.

In Experiment 1, a one-way repeated measures ANOVA and *post hoc* multiple comparison with the Bonferroni correction were performed for comparison on Hit rate among target speeds. And, the paired *t*-test was conducted to compare between Hit and Miss on the SEA and RCA at 5% significance level. Regression analysis of the relationship between hit rate and SEA incidence (Figure 3B) and the relationship between hit rate and SEA and RCA co-occurrence rate (Figure 3C) was performed by segmented regression and simple regression, respectively. In Experiment 2, the paired *t*-test was used to compare the control and NoFB conditions at 5% significance level.

## Results

We examined how gaze behavior is conducted during continuous visuomotor actions and contributes to the performance using the CVM task.

### Characteristics of the Gaze Movement in the Vertical Direction of the LC Display

Figures 2A,B show typical examples of the trajectory of the eye and the cursor positions on the vertical axis of the LC display at a target speed of 3,000 pixels/s. The participant's gaze moved typically in a saccadic manner to the zero value (the vertical position of the target's center) in a few hundred milliseconds after target appearance. This movement represented the SEA (orange line in Figures 2C,D,F). Like the SEA, the cursor movement that rapidly approached the Hit zone was called the RCA (green line in Figures 2C,E,F). Both the SEA and RCA were commonly observed regardless of the target speed in the task.

### Relationship Between the Task Performance and Gaze Movements

Figure 3A depicts the Hit rate and SEA occurrence rate plotted against the target speed. Both curves declined in a sigmoidal manner in the same target speed range, but the shape of the two curves differed from each other.

The Hit rate curve began to decline at target speeds over 3,000 pixels/s, then became linear, and finally plateaued at 5,000 pixels/s. One-way ANOVA showed that there was a main effect for the target speed condition (*F*_(6,120)_ = 390.8, *p* < 0.01, η^2^ = 0.92), and the Bonfferoni correction showed that the difference between 2,000 and 3,000 pixels/s was significant (*p* < 0.01), but not significant between 1,000 and 2,000 pixels/s (*p* = 0.74), between 5,000 and 6,000 pixels/s (*p* = 1.0). Therefore, the target speed condition can be divided by two inflection points (*2*,000 pixels/s and 5,000 pixels/s) into three categories: low (1,000–2,000 pixels/s), middle (3,000–4,000 pixels/s), and high (5,000–7,000 pixels/s) speeds, which were characterized by a high Hit rate, a speed-dependent decline in the Hit rate, and a speed-independent low Hit rate (a constantly high frequency of Miss), respectively.

In the present study, not only the cursor but also the gaze approached the Hit zone in the CVM task. Both the SEA occurrence rate and Hit rate curves began to decline beyond a target speed of 3,000 pixels/s, suggesting the functional role of the SEA in the task performance. To examine this point further, we investigated the relationship of the Hit rate and the SEA occurrence rate (Figure 3B) and that of the Hit rate and the SEA and RCA co-occurrence rate (Figure 3C). Regression analysis was performed using data from all target speeds for all participants. The Hit rate plotted against SEA occurrence rate was fitted by two linear regression lines that connected at a SEA occurrence rate of about 60% (*r* = 0.83, *p* < 0.01, Figure 3B). In other words, the Hit rate was low (around 25%) as long as the SEA occurrence rate was below 60%. The SEA and RCA co-occurrence rate also correlated strongly with the Hit rate in a linear manner (*r* = 0.87, *p* < 0.01, Figure 3C). Thus, the expression of the SEA seems to be an important factor in the success of a task in association with the occurrence of the RCA.

The hypothesis to be tested in this study was that “(1) predictive saccades are involved in continuously striking fast-moving balls (targets) under time-constrained conditions such as table tennis, and (2) the performance of the saccade contributes to the success or failure of the striking and the accuracy of the strike performance.” From the results in Figure 3A, it was found that the target speed conditions suitable for testing this hypothesis are 3,000 pixels/s and 4,000 pixels/s, where the Hit rates were neither too high nor too low. Therefore, we will focus on these two target conditions and proceed with the following analysis. To examine whether and how the gaze behavior was associated with the task performance, the spatiotemporal parameters of the SEA were analyzed for Hit and Miss trials separately.

### Gaze Movements Contributing to RCA Performance

**Figure 4:**
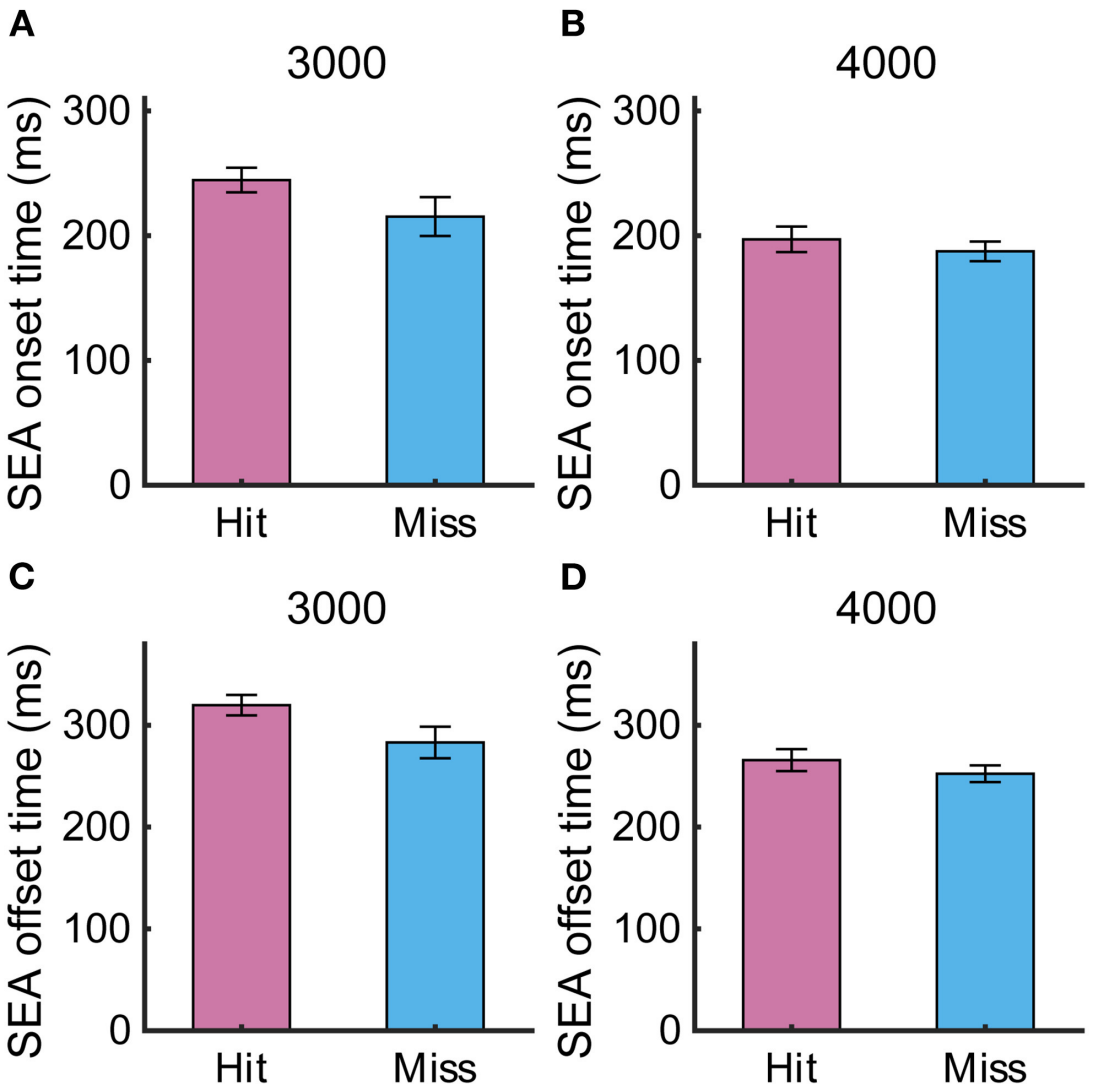
**(A,B)** SEA onset time and **(C,D)** offset time at target speeds of 3,000 and 4,000 pixels/s in Experiment 1. Pink and light blue bars show Hit and Miss, respectively. Error bars are SEM.

To examine whether the temporal parameters of the SEA were related to the task performance, we compared the SEA onset time and offset time between Hit and Miss trials (Figure 4), but found no significant difference (SEA onset time: 3,000 pixels/s, 244 ± 9.8 ms vs. 215 ± 15.5 ms, *p* = 0.10; 4,000 pixels/s, 197 ± 10.2 ms vs. 187 ± 7.8 ms, *p* = 0.50; SEA offset time: 3,000 pixels/s, 319 ± 9.9 ms vs. 283 ± 15.5 ms, *p* = 0.06; 4,000 pixels/s, 265 ± 10.7 ms vs. 252 ± 8.2 ms, *p* = 0.31), suggesting that the such temporal properties of the SEA were not directly associated with the task performance. There was no difference in onset time between SEA and RCA, but we found that SEA offset time was significantly shorter than RCA offset time (Supplementary Figure 1). This means that the cursor is still moving when the acquisition of visual information is resumed after the SEA is completed, suggesting the possibility of correcting the cursor movement by the obtained information.

**Figure 5:**
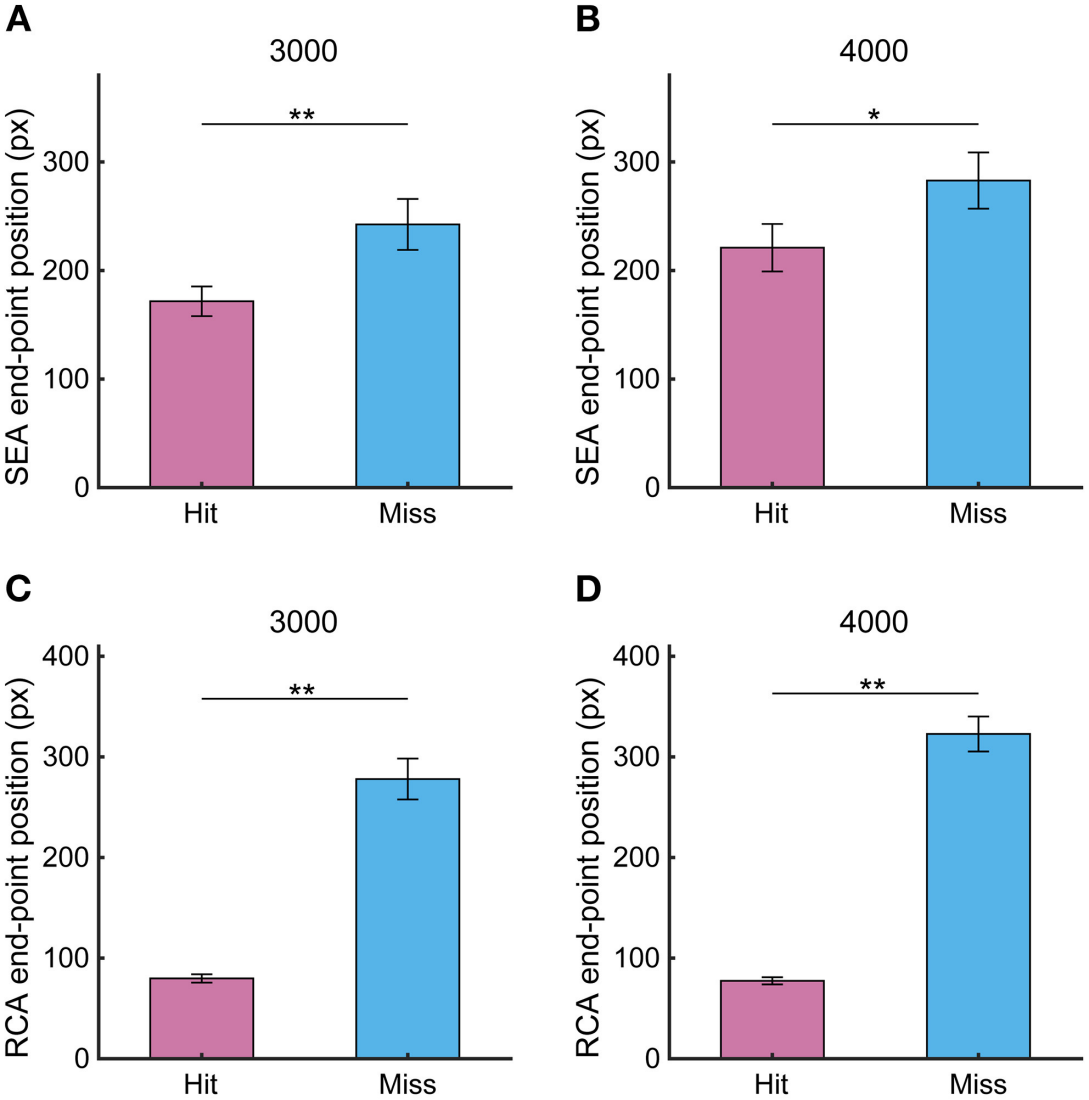
The end-point position of **(A,B)** SEA and **(C,D)** RCA at target speeds of 3,000 and 4,000 pixels/s in Experiment 1. Pink and light blue bars show Hit and Miss, respectively. Error bars are SEM. *p < 0.05, **p < 0.01.

Since the spatial resolution of the visual field gets worse from the central field to the periphery, the SEA end-point position determines the quality of the visual information that is acquired after the SEA and used to control the RCA movement as a feedback signal. Therefore, we first investigated the percentage of SEA that the gaze reached the Hit zone, finding 57.8 ± 5.0 % for 3,000 pixels/s and 48.5 ± 4.7 % for 4,000 pixels/s. Since SEA end-point position was out of the Hit zone in almost half trials, we calculated the percentage of SEAs that ended before reaching the Hit zone to the total number of SEAs that ended outside the Hit zone, showing that almost all the SEAs out of the Hit zone ended before it (3,000 pixels/s: 96.6%; 4,000 pixels/s: 97.8%). Next, we compared the SEA end-point position between Hit and Miss trials and found that the SEA end-point position was significantly closer to the Hit zone in the Hit trial than in the Miss trial (Figures 5A,B, 3,000 pixels/s, *p* < 0.01; 4,000 pixels/s, *p* < 0.05). The difference in the SEA end-point position between the Hit and Miss trials was 70.8 ± 23.4 pixels at 3,000 pixels /s and 57.3 ± 21.2 pixels at 4,000 pixels /s. The end-point position of the RCA showed similar results as the SEA, as it was significantly smaller in Hit trials than Miss trials (Figures 5C,D, 3,000 pixels/s, *p* < 0.01; 4,000 pixels/s, *p* < 0.01). The difference in the RCA end-point position between the Hit and Miss trials was 198.2 ± 20.9 pixels for 3,000 pixels/s and 241.8 ± 21.1 pixels for 4,000 pixels/s.

**Figure 6:**
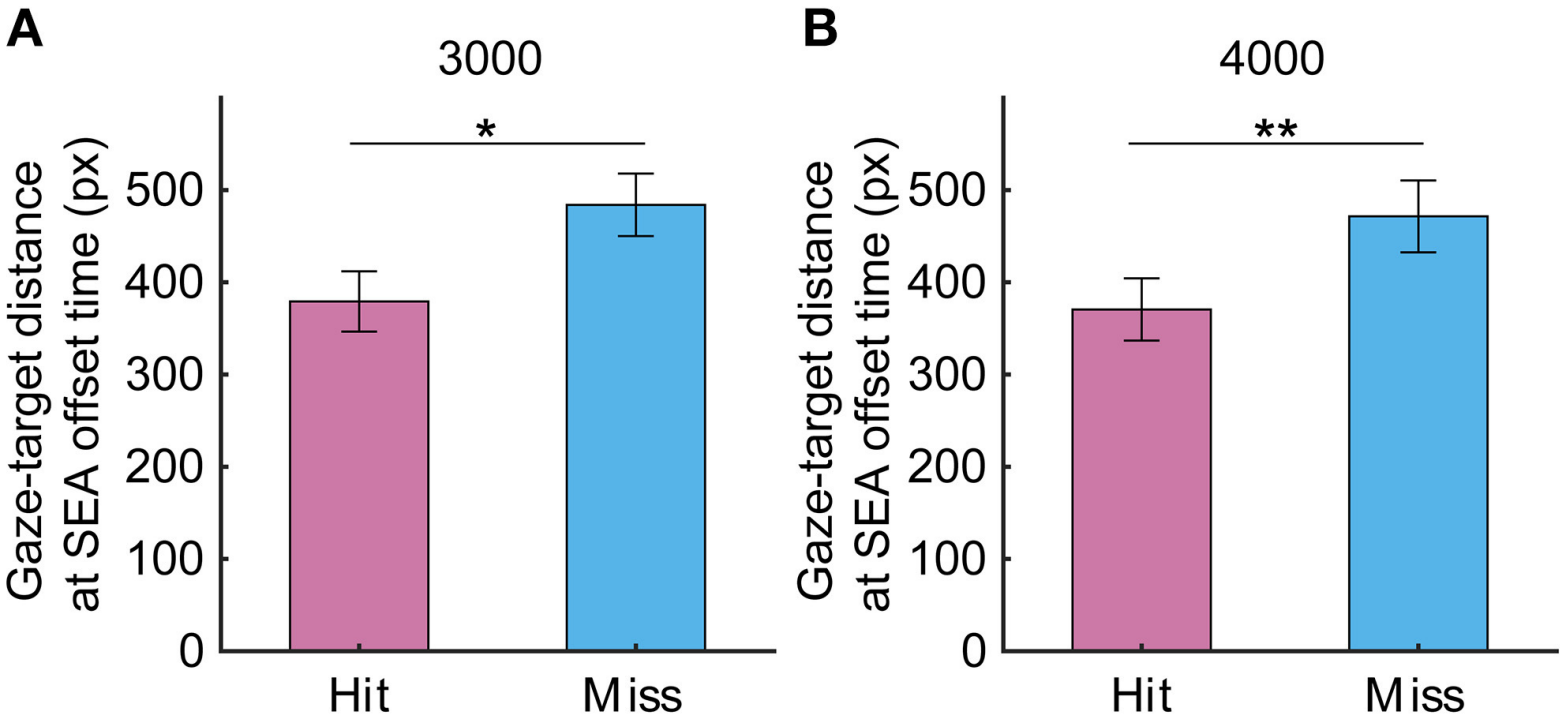
The gaze target distance at target speeds of **(A)** 3,000 and **(B)** 4,000 pixels/s in Experiment 1. Pink and light blue bars show Hit and Miss, respectively. Error bars are SEM. *p < 0.05, **p < 0.01.

To investigate in more detail how SEA moved during the CVM task, the X-axis position of gaze relative to the X-axis position of the target center was calculated at the onset time and offset time of the SEA. At the start of SEA, the gaze was located about 400 to 600 pixels to the left of the target, and the distance was reduced to <300 pixels at the end of SEA, suggesting that the gaze moved toward the predictive arrival point of the target (X-axis position at SEA onset time: 3,000 pixels/s, −582.6 ± 68.2 pixels vs. −679.2 ± 90.9 pixels, *p* = 0.19; 4,000 pixels/s −476.1 ± 78.6 pixels vs. −522 ± 79.1 pixels, *p* = 0.31; X-axis position at SEA offset time: 3,000 pixels/s, −167.8 ± 53.6 pixels vs. −285.9 ± 60.1 pixels, *p* < 0.05; 4,000 pixels/s−24.4 ± 63.2 pixels vs. −70.7 ± 66.5 pixels, *p* = 0.24). So next, we investigated whether gaze-target distance, that is, the distance from gaze position to target center is associated with the success or failure of the task (Figure 6). As a result, the gaze-target distance at SEA offset time was significantly smaller in the Hit trials than the Miss trials (3,000 pixels/s, 379 pixels for Hit, 484 pixels for Miss, *p* < 0.05; 4,000 pixels, 370 pixels for Hit, 471 pixels for Miss, *p* < 0.01). These results suggest that information related to the spatial accuracy of SEA reaching the target may enhance the spatial accuracy of RCA.

### SEA-Related Information Responsible for RCA Performance

There are three types of SEA-related information that may regulate RCA: visual information from the retina, proprioceptive information derived from the extraocular muscles, and efference copy of the eye movements. Any of these types may explain the relationship between the end-point positions of the SEA and RCA.

**Figure 7:**
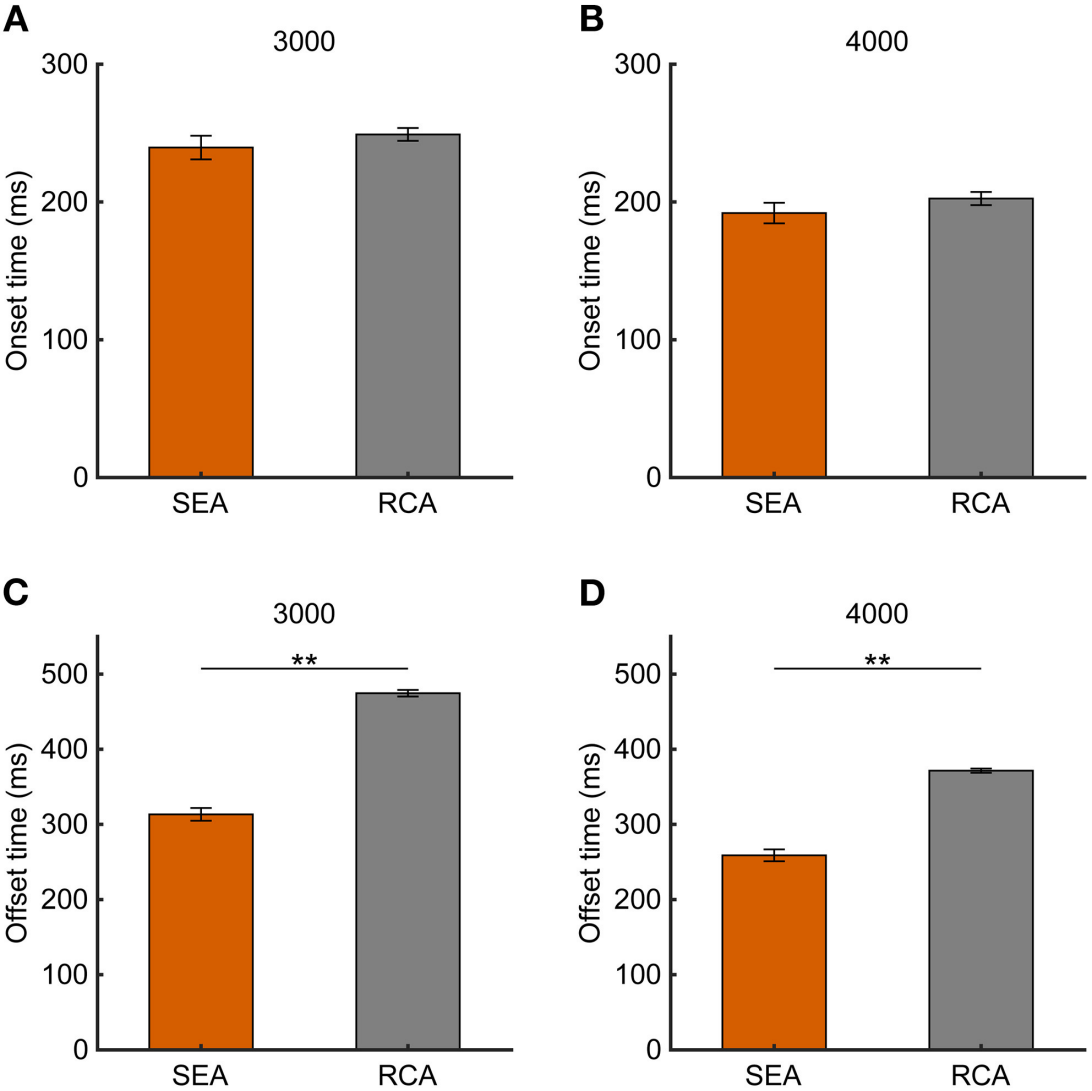
**(A,B)** Onset time and **(C,D)** offset time at target speeds of 3,000 and 4,000 pixels/s. Orange and gray bars show SEA and RCA, respectively. Error bars are SEM. ^**^p < 0.01.

If the SEA-related information contributes to the RCA, then the SEA should have finished before the RCA. To confirm this point, the onset time and offset time were analyzed for the SEA and RCA. Although no difference in the onset time between the SEA and RCA was observed (Figures 7A,B, 3,000 pixels/s, 249 ± 4.6 ms vs. 239 ± 8.5 ms, *p* = 0.18; 4,000 pixels/s, 202 ± 4.8 ms vs. 191 ± 7.4 ms, *p* = 0.29), the SEA offset time was earlier than the RCA offset time due to the speed of the saccades (Figures 7C,D, 3,000 pixels/s, *p* < 0.01; 4,000 pixels/s, *p* < 0.01).

To test the first of the three possibilities, we conducted the target disappearance task, in which the target was made invisible for 100 ms from the end of the SEA (NoFB). If the first possibility of the visual feedback control is the cause, then the distance of the RCA end-point position from the Hit zone should increase with the disappearance of the target. If not, the RCA end-point position should be unchanged.

**Figure 8:**
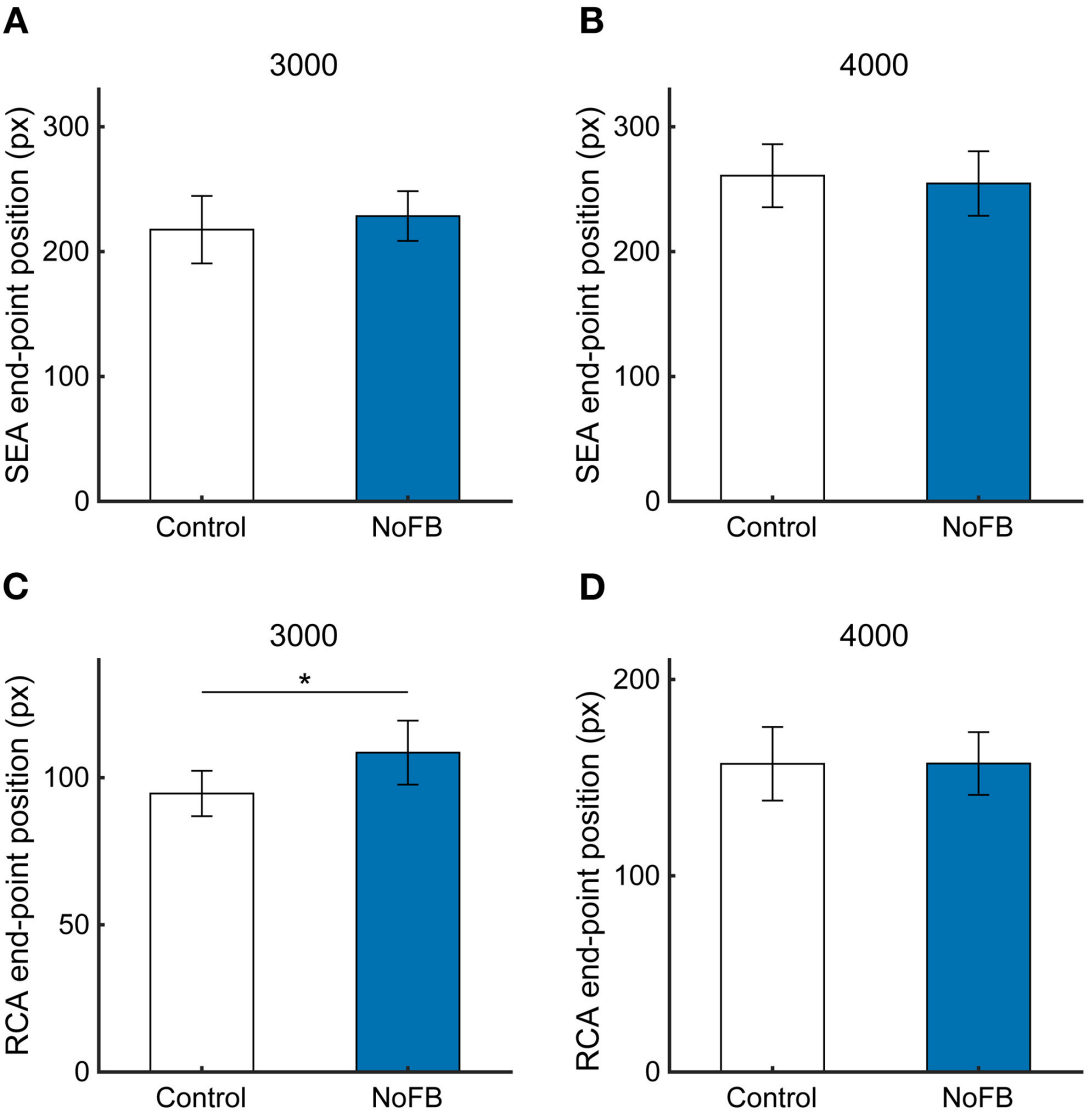
The end-point positions of **(A,B)** SEA and **(C,D)** RCA at target speeds of 3,000 and 4,000 pixels/s in Experiment 2. White and blue bars show control and NoFB conditions, respectively. Error bars are SEM. *p < 0.05.

Typical cursor and gaze trajectories during the task under NoFB conditions at a target speed of 3,000 pixels / s are shown in Supplementary Figure 2. Figure 8 shows the SEA and RCA end-point positions at 3,000 and 4,000 pixels/s. The SEA end-point position was not influenced by the target disappearance, which is as expected, because the target disappeared after the SEA ended (Figures 8A,B, 3,000 pixels/s, 217 ± 27.0 pixels vs. 228 ± 19.9 pixels, *p* = 0.59; 4,000 pixels/s, 260 ± 25.2 pixels vs. 254 ± 25.8 pixels, *p* = 0.81). On the other hand, the RCA end-point position at 3,000 pixels/s was significantly larger in NoFB than control (Figure 8C, *p* < 0.05), but the RCA end-point position at 4,000 pixels/s was not different (Figure 8D, 157 ± 18.7 pixels vs. 157 ± 15.9 pixels, *p* = 0.99). These results suggest that the online feedback control based on visual information just after the saccade can be executed in a continuous visuomotor reaction.

## Discussion

Gaze behavior in continuous visuomotor actions to a fast-moving target is not well understood. In the present study, we investigated how eye movement contributed to the performance of a psychophysiological task. The main results are summarized as follows: (1) eyes typically moved rapidly toward the Hit zone in a saccadic manner, (2) the co-occurrence rate of the SEA and RCA was strongly correlated with the task performance (Hit rate), (3) the SEA was completed before completion of the RCA, (4) the SEA and RCA end-point positions in the Hit trials were closer to the Hit zone than in the Miss trials, and (5) the RCA end-point position was larger in the NoFB condition than control condition.

### Spatiotemporal Relationship Between Gaze and Cursor Movements

We found that the end-point position of the RCA, which is a ballistic cursor movement approaching the Hit zone, is an important factor in the task performance. We also found that the gaze moved toward the Hit zone in a saccadic manner like the cursor but was completed first. Interestingly, the occurrence of the SEA was associated with the Hit rate, and the co-occurrence rate of the SEA and RCA correlated with the Hit rate, suggesting a potential functional role of the SEA in the cursor movement. Since neither the SEA onset time nor offset time differed between the Hit and Miss trials, temporal factors of the SEA do not seem to be a determinant of the task performance. However, the SEA end-point position and the gaze-target distance at SEA offset time were remarkably different between Hit and Miss trials (see Figures 5A,B, 6) and was associated with the RCA end-point position (see Figures 5C,D). This observation suggests that the spatial factor of the SEA end-point position is related with that of the RCA end-point position.

### Motor Correction at the Ballistic Movement

Our results suggest that the RCA was adjusted on-line based on the visual information. The RCA is a ballistic movement executed within a few hundred milliseconds. Such ballistic movements have been previously considered to be expressed as feedforward control only. However, arm reaching movement, which is representative ballistic muscular movement, has been reported to be modified on-line (Soechting and Lacquaniti, 1983; Prablanc and Martin, 1992; Brenner and Smeets, 1997; Kadota and Gomi, 2010). For example, when the target (destination of reaching) jumped to another position after the arm reaching movement began, the trajectory of the fingertip was corrected toward the new target position after a certain latency (Prablanc and Martin, 1992; Day and Lyon, 2000; Kadota, 2010). This motor correction occurred usually within 170 ms after the target moved and was even observed within 100 ms in some cases. Interestingly, Prablanc and Martin (1992) discovered that reaching movement is implicitly corrected on the basis of on-line visual information. Even if the target jumped to a new location during a saccade in which the brain processing of visual information was suppressed (saccadic suppression), the movement path was corrected toward the new target location using visual information obtained after saccades ended. The participant did not perceive the target jump, suggesting that the motor was corrected on the basis of updated visual information obtained after the end of the saccade. Thus, on-line motor correction is automatically conducted, a phenomenon known as automatic pilot, and visual feedback plays an important role in controlling the ballistic arm movement. Those previous findings support the possibility that the ballistic cursor movement of the RCA was controlled by visual feedback information for the target obtained after the end of the SEA in the present study.

We considered three possible feedback and feedforward controls through which the SEA contributes to task performance: feedback control based on visual information concerning the position difference between the cursor and target; feedback control based on proprioceptive information derived from the ocular muscles that produce the SEA; and RCA control due to the efference copy issuing feedforward control from the center of the eye movement to produce a motor command to the ocular muscles. Using the target disappearance task, we demonstrated that visual feedback information can be used to direct the cursor toward the Hit zone through an on-line motor correction. Since visual information processing during the saccade is inhibited by saccadic suppression, the acquisition of visual information for feedback control was considered to start after the completion of the saccadic eye movement to the Hit zone (i.e., SEA). In the target disappearance task, the target was made invisible just after the end of the SEA, increasing the distance between the end-point position of the cursor and the Hit zone. Therefore, visual information of the target can be acquired and used on-line for motor correction via feedback control.

Regarding the second and third possibilities, the RCA is corrected by proprioceptive information originating from the ocular muscle or motor information from the efference copy of the SEA. Although these motor control pathways are unaffected by the target disappearance after the end of the SEA, the distance of the RCA end-point position from the Hit zone was observed to have enlarged. Therefore, ballistic cursor movement seems to be controlled by visual information from the retina acquired after saccades when visual feedback is available at a target speed of 3,000 pixels/s. On the other hand, the motor correction also might be performed by saccade-related information except for retinal one since the distance between the end-point position of the cursor was unaffected by the target disappearance even if visual feedback is unavailable at a target speed of 4,000 pixels/s.

From the results of this study, it is possible to infer how the control mechanism of continuous reaching movement changes as the target speed increases, that is, the time constraint becomes stricter. Reaching movement is initiated by feedforward control by motor commands, but is amended/regulated by retinal and non-retinal information related to predictive saccades, realizing high accurate reaching. As the time constraint becomes stricter, first, control by retinal information becomes impossible, then control by non-retinal information, and finally, feedforward control becomes impossible. The present study is the first report in the world of the contribution of multiple motor control mechanisms related to saccades to continuous visuomotor performance. This information is considered valuable for systematically understanding the human motor control system and for exploring ways to avoid motor errors.

### Neural Mechanism of Visuomotor Performance Improvement by Predictive Saccades Toward a Target

This study suggested that the spatial accuracy of the predictive saccades toward the target during the task is related to the spatial accuracy of the cursor movement. The relationship is thought to be caused by the spatial accuracy of the visual information obtained after the saccade. When the gaze-target distance at the end of SEA is calculated as the viewing angle in consideration of the eyeball position and the gaze position, it corresponds to about 10 degrees in the Hit trial, meaning that the target was projected on the macular region with a high spatial resolution (perifovea, viewing angle of 9.2 degrees). On the other hand, in the Miss trial, it corresponds to about 14 degrees, which means that the target was captured by extra-fovea region outside the macula (Polyak, 1941; Osaka, 1983). Therefore, at target speed 3,000 pixels/s, where the retinal information acquired after SEA may contribute to the improvement of RCA spatial accuracy, whether the retinal information is derived from the intramacular or extramacular region may determine the success or failure of the task.

However, it is premature to conclude that only the spatial resolution of visual information contributes to the spatial accuracy of cursor movement. It has been reported that in the reaching movement of the hand, the network of brain regions activated depends on whether the eye directly sees the target before reaching it (Prado et al., 2005). Reaching while looking at the target in the central visual field activated the local network (medial intraparietal sulcus and caudal part of the dorsal anterior area). On the other hand, when reaching the target in the peripheral vision, a wider network including the parietal-dorsal junction (POJ) was activated. Therefore, the spatiotemporal characteristics of information processing in these networks may ultimately contribute to the spatial accuracy of cursor movement. Further research is needed to clarify this point.

On the other hand, at a target speed of 4,000 pixels/s, where non-retinal information related to saccades can contribute to improved RCA spatial accuracy, the information of gaze movement is important to know the target Y-axis position. Therefore, the information in the Y-axis direction of the gaze, especially, the Y-axis information of the end-point position of the saccade, may be an important clue to know the Y-axis position of the target. In the Hit trial at 4,000 pixels/s, the SEA end-point position was about 220 pixels, 80 pixels away from the hit zone boundary. This distance corresponded to a visual angle of 2.7 degrees in this experiment. On the other hand, the SEA end-point position for the Miss trial was about 280 pixels, 140 pixels away from the Hit zone boundary, corresponding to a visual angle of 4.7 degrees. The difference in the SEA end-point position between the Hit and Miss trials may reflect the spatial accuracy for the proprioceptive information originating from the ocular muscle or for the motor information of the efference copy of SEA, possibly making the difference in correcting the cursor movement.

### Gaze Behavior in Sports

Previous studies have investigated how eye movements of athletes while playing sports are related to sports performance or skill, finding the importance of both smooth pursuit and saccade eye movements. For example, superior batters in baseball and cricket were found to maintain smooth pursuit of the ball longer than inferior batters (Hubbard and Seng, 1954; Kishita et al., 2020) and to have a higher percentage of the pursuit occupied in the total eye movement. The efference copy generated with the motor command for the pursuit eye movement provides information of the motion direction and speed of the visual target, such as a ball, to predict the trajectory and future location of the target (Spering et al., 2011). Therefore, it is expected that the more accurate the pursuit, the more accurate the prediction. However, there is an upper limit of about 40–50 deg/s in pursuit eye movement speed (Buizza and Schmid, 1986), and athletes have been known to track the ball by not only pursuit but also by saccades in real sports scenes, such as baseball, cricket, and table tennis. The gaze of highly skilled table tennis players was reported to not follow the whole trajectory of the ball but rather was directed with saccades to the opponent's launch point in the early phase and to the predicted collision point of the racket and the ball in the swinging phase (i.e., performing visuomotor coordination) (Rodrigues et al., 2002). The predictive saccades to the predicted hitting point was commonly observed in the present study. Therefore, it is possible that the visual information obtained just after the predictive saccades is used to amend ball-hitting movements in real table tennis scenes too.

Our findings suggest the importance of directing the gaze to the ball on the way that the racket/bat is approaching a moving target when hitting the ball in a sports scene. The essence of the instruction “swing the racket / bat while looking at the ball”, which is often said in the sports scene, is profound and reasonable from a neuroscientific point of view.

## Data Availability Statement

The original contributions presented in the study are included in the article/Supplementary Material, further inquiries can be directed to the corresponding author.

## Ethics Statement

The studies involving human participants were reviewed and approved by the Ethics Committee of the Graduate School of Medicine, Osaka University. The patients/participants provided their written informed consent to participate in this study.

## Author Contributions

CA and SS designed the research, collected the data, performed the analyses, and wrote the paper. NS provided technical assistance for the task development. KK, YY, and RG contributed to planning of this research. All authors contributed to the article and approved the submitted version.

## Funding

This work was financially supported by KAKENHI (Grant Nos. JP16H01869, JP16K12996, and 25560302 to SS, and JP19K19977 to CA) and the Sports Research Innovation Project (SRIP) Grant to SS and Grant-in-Aid for JSPS Fellows (JP17J08456) to CA.

## Conflict of Interest

The authors declare that the research was conducted in the absence of any commercial or financial relationships that could be construed as a potential conflict of interest.

## Publisher's Note

All claims expressed in this article are solely those of the authors and do not necessarily represent those of their affiliated organizations, or those of the publisher, the editors and the reviewers. Any product that may be evaluated in this article, or claim that may be made by its manufacturer, is not guaranteed or endorsed by the publisher.
