# Supplementary material for: Spatial Accuracy of Predictive Saccades Determines the Performance of Continuous Visuomotor Action
Source: Front Sports Act Living. 2022 Jan 17;3:775478. doi: 10.3389/fspor.2021.775478 (PMC8801910; doi:10.3389/fspor.2021.775478)
Supplement: Supplementary file 1 [file Image_1.PDF]

Supplementary Material

1 Supplementary Figures

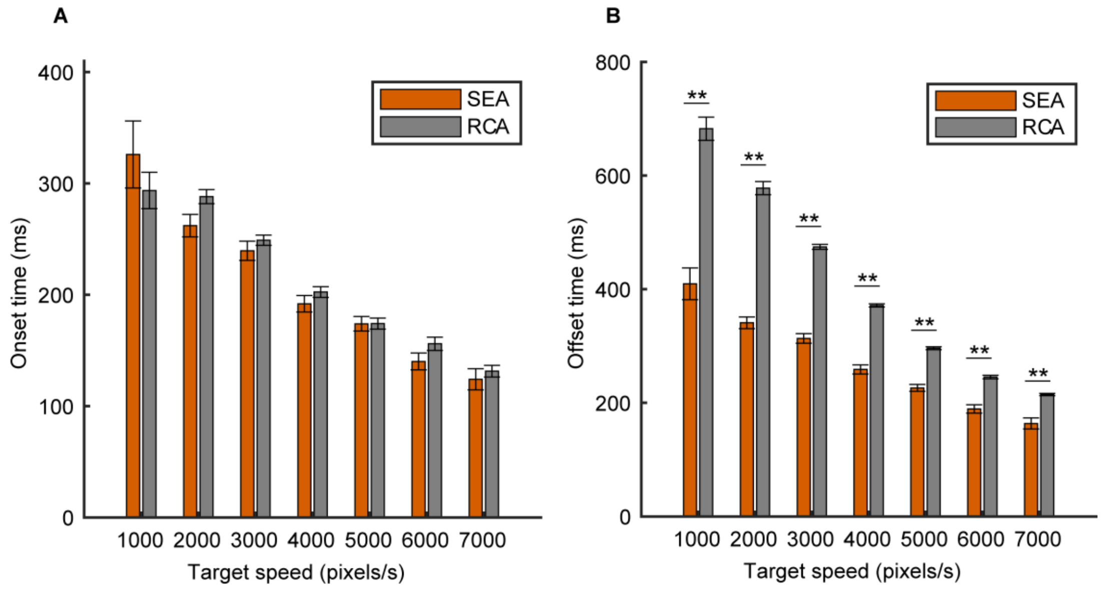

Supplementary Figure 1. (A) Onset time and (B) offset time. Orange and gray bars show SEA and RCA, respectively. Error bars are SEM. **p < .01.

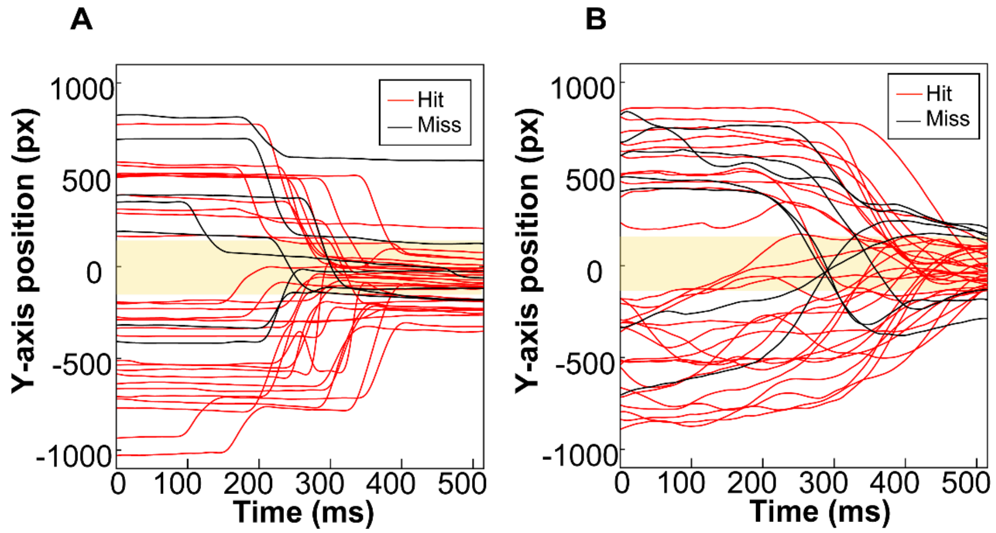

Supplementary Figure 2. Typical cursor and eye trajectories during the task under the NoFB condition. (A) All trial trajectories of eye movements to the vertical axis on the display at the target speed 3000 pixels/s. (B) All trial trajectories of the cursor movement to the vertical axis on the display at the target speed 3000 pixels/s. Red and black lines indicate Hit and Miss trials, respectively.
